# Breaking Bad News in Ethnic Settings: Perspectives of Patients and Families in Northern Sri Lanka

**DOI:** 10.1200/JGO.2016.005355

**Published:** 2016-08-10

**Authors:** Chrishanthi Rajasooriyar, Jenny Kelly, Thanikai Sivakumar, Gowcikan Navanesan, Shahini Nadarasa, Madona Hashanthy Sriskandarajah, Sabe Sabesan

**Affiliations:** **Chrishanthi Rajasooriyar** and **Madona Hashanthy Sriskandarajah**, Jaffna Teaching Hospital; **Chrishanthi Rajasooriyar**, Tellipalai Trail Cancer Hospital, Jaffna; **Thanikai Sivakumar** and **Shahini Nadarasa**, National Hospital of Sri Lanka; **Gowcikan Navanesan**, Teaching Hospital, Karapitiya, Sri Lanka; **Jenny Kelly** and **Sabe Sabesan**, Townsville Hospital and Health Service; **Jenny Kelly** and **Sabe Sabesan**, James Cook University; and **Sabe Sabesan**, The Townsville Hospital, Townsville, Queensland, Australia.

## Abstract

**Purpose:**

The discussion of a cancer diagnosis and prognosis often is difficult. This study explored the expectations of Tamil-speaking patients with cancer and their families with respect to receiving their cancer diagnosis in northern Sri Lanka.

**Methods:**

This exploratory, descriptive, qualitative study used semistructured interviews.

**Results:**

Thematic analysis identified two major themes: communication and information seeking. The findings illustrate a discrepancy between patient preference for direct disclosure of the diagnosis and that of families. Ninety-five percent of patients wanted medical staff to disclose their cancer diagnosis, whereas only 45% of family members believed that the diagnosis should be disclosed to the patient rather than to the family.

**Conclusion:**

Although patients and their family members’ views and expectations of the disclosure of diagnosis and prognosis differ, a majority of patients want to be told directly about their diagnosis rather than to learn of it from a relative. The findings are similar to the literature on other ethnic groups from Sri Lanka and studies from English-speaking developed countries. Therefore, the main questions are how to educate families and physicians about the benefits of open disclosure to patients and how to change culture. Results of this study along with a previous study call for the development of strategies and guidelines to improve societal views, educate patients and families, and train health professionals in the area of breaking bad news and discussing prognosis in the Sri Lankan setting.

## INTRODUCTION

The issue of breaking bad news to patients has been a key topic in the literature for many years. Discussion of prognosis and end-of-life issues often is difficult, and research has identified deficiencies in communication between health care professionals and patients on this aspect of clinical care.^1^ Many health care professionals are uncomfortable with discussing these topics for many reasons, including perceived lack of training, stress, lack of time to attend to the patient’s emotional needs, fear of upsetting the patient, and a feeling of inadequacy or hopelessness with regard to the unavailability of further curative treatment.^2-4^ Such avoidance can result in poor patient satisfaction, psychologic morbidity, and poor treatment decisions.^5-7^ Therefore, the patient’s interests are best served by offering such information rather than withholding it in an attempt to protect the patient from losing hope or being upset.^8^

Although the global trend toward disclosing the truth to patients is increasing,^9^ and disclosure may be considered an important practice today in Western countries, this is not the same for many non-Western countries.^10^ These practices vary among countries, cultures, religions, and social backgrounds.^11,12^ In some cultures, patients do not want to know their diagnosis and their families do not want them to know their diagnosis.^9^ In some countries, such as China, direct disclosure is now required by law.^10^ Developed countries such as the United States had a similar practice of nondisclosure five decades ago.^13^ However, this practice has changed rapidly during the last quarter of the century, and breaking the bad news to the patient has become an important aspect of patient care.^14,15^

Similar to many non-Western countries, physicians in Sri Lanka face issues related to breaking bad news. They also face controversial questions such as whether to tell, to whom, and when. Currently, no consensus-based national guidelines have been developed for breaking bad news and discussing prognosis in Sri Lanka.

Many ethnic groups live in Sri Lanka, including Sinhalese (74%) and Tamils (18%) who speak Sinhalese and Tamil and practice mostly Buddhism and Hinduism, respectively. The Tamil minority mostly resides in the Eastern and Northern Provinces. Conflict between the Tamils and the Sinhalese resulted in civil war, which lasted three decades and resulted in the death of thousands of Tamils and an exodus of many more to other countries around the world. The Sri Lankan diaspora numbers approximately 3 million worldwide, with significant communities now settled in Europe, the United States, Asia, and Australasia. Given the population of 21 million, one in every eight Sri Lankans are based overseas, which makes a remarkable diaspora-to-population ratio not matched by any of the country’s south Asian counterparts.^16^ In the Northern Province of Sri Lanka, family bonding is strong, and people typically live with extended family members in one large household. Anecdotally, when a patient is given a diagnosis of cancer, the family prefers to hide the diagnosis from the patient in an attempt to protect him or her from perceived emotional harm. The family usually instructs the physician not to disclose the information directly to the patient. Nonconveyance of a cancer diagnosis to the patient has become common practice, except for rare occasions where patients may insist on knowing their diagnosis. Because no national guidelines or consensus exist on breaking bad news, this study explored the expectations of Tamil (the dominant ethnic group in northern Sri Lanka)-speaking patients with cancer and their families with respect to receiving their cancer diagnosis.

## METHODS

### Study Design

This study used a qualitative approach to explore the experiences and expectations of patients with cancer and their family members in northern Sri Lanka. Respondents who spoke Tamil as their first language were selected through purposive sampling. Semistructured interviews were used to explore patient and their nominated family member’s expectations and experiences of receiving their cancer diagnosis and views on the extent of information sought for future management of and dealing with the cancer.

### Study Setting

The study was conducted at a major cancer teaching hospital in the Northern Province of Sri Lanka. Ethical approval was received from the Faculty of Medicine, Ethics Review Committee, University of Jaffna. No funding was provided to this project.

Four junior physicians (1 year postgraduation; three female) conducted semistructured interviews with patients and their family members with the use of a flexible interview guide. The research team developed the interview guide and piloted and adapted it on the basis of local feedback. In addition to demographic questions, the interview guide queried about the type and nature of cancer, amount and timing of information, and who should be told the diagnosis first.

The interviewers worked in pairs, and all interviews were conducted in Tamil. The interviewers have never worked in cancer wards and did not know the patients or their families. They received qualitative research training by an experienced Australian nurse researcher. The training was conducted through Skype and included a number of mock interviews, which allowed for detailed feedback before conducting the actual interviews. Also before the interviews, respondents read a plain-language statement that explained the aim of the study and the implications of the results to inform physicians on breaking bad news in the future and signed a written consent form.

Patients and their family members were asked slightly different questions, but all respondents were asked questions on the way the patient was or was not told about their cancer diagnosis and how the patient and family member felt about this process. Forty face-to face interviews were conducted: 20 with patients and 20 with their nominated family members. Four patients were selected from each of the five districts of the Northern Province to have a fair representation of respondents from the entire province. Data saturation was reached after 10 interviews. To focus mainly on the issue of breaking bad news without confusing the respondents with discussing prognosis, patients with metastatic disease were excluded. One patient refused because of the distance he had to travel, and another patient believed that he could not talk continuously in an interview due to treatment-induced fatigue. The interviews were conducted in the clinic rooms mostly during weekends or in the afternoons when no clinics were scheduled. Interviews were digitally recorded with respondents’ permission and transcribed verbatim. Patient interviews took between 17 and 55 minutes (min), with an average interview time of 24 min. Family member interviews were a little shorter and ranged between 15 and 30 min, with an average interview time of 21 min. Patients and their family members were interviewed separately to allow for frank and open discussion. All interviews were conducted in Tamil and translated into English by a certified translator and sent to the Australian researcher for assistance with coding and analysis. Handwritten notes were also taken, and at the end of each interview, these notes were read aloud to the respondent to check for accuracy and clarify any outstanding issues.

### Data Analysis

Iterative thematic analysis was used to examine the qualitative data. The transcripts were analyzed by using an inductive coding approach.^17^ Open codes were extracted from the transcript by one of the researchers and grouped together manually in Excel tables (Microsoft Corporation, Redmond, WA) to form preliminary themes and subthemes. Two researchers compared and discussed codes and reached consensus on the themes.

## RESULTS

Twenty patients with cancer and 20 nominated family members participated in face-to-face interviews. Patient characteristics are presented in Table 1. All patients were Sri Lankan Tamils. Of the 20 family members, 19 were married with children, with a median age of 35 years. One member was a parent of a younger patient. Thirty percent of respondents had a postgraduate degree, and 50% had completed Graduate Certificate of Education Advanced Level (grade 12 high school). Analyses identified the same two major themes for patients and their families—communication and information seeking—and therefore are not reported separately.

**Table 1:**
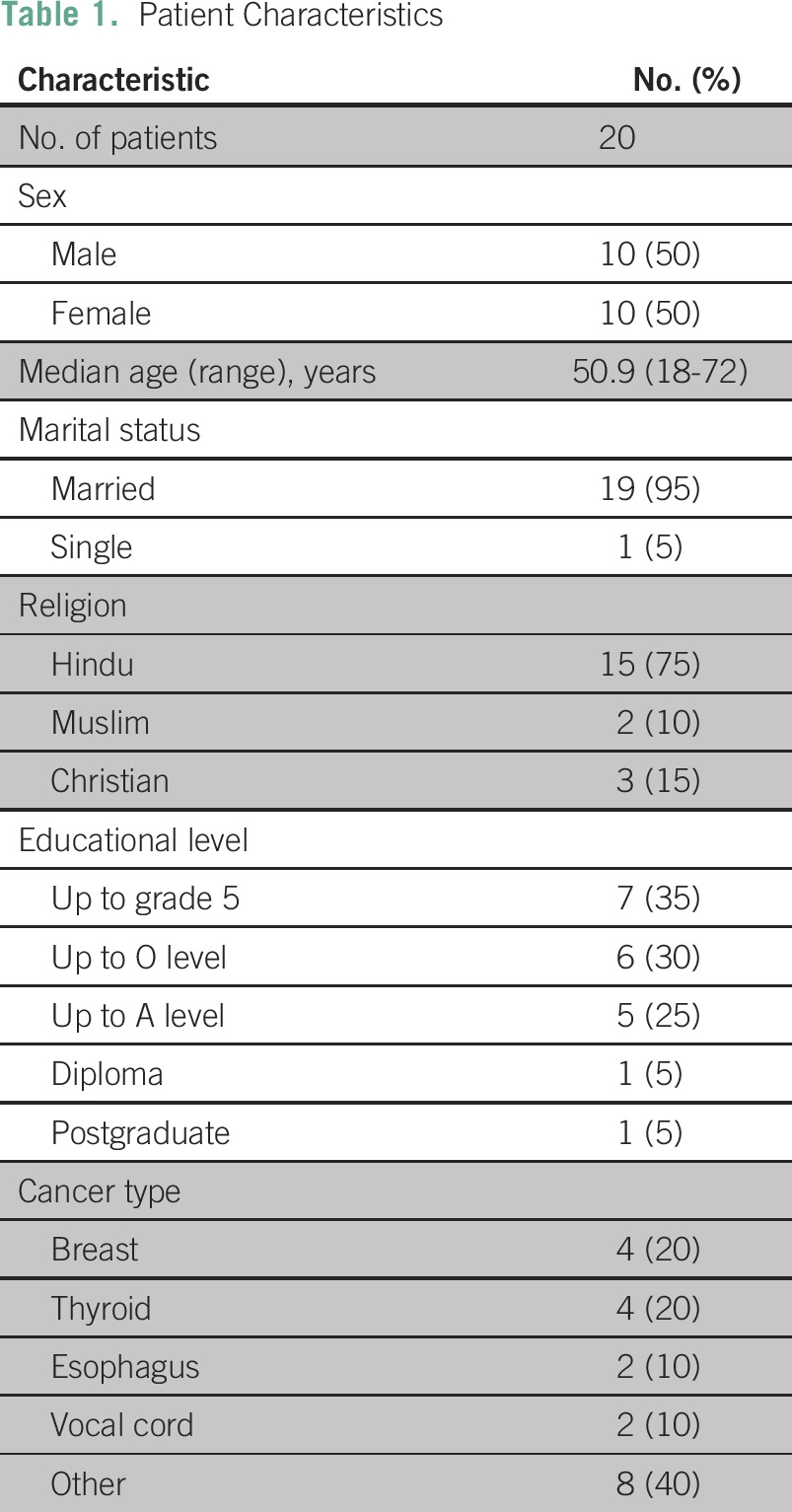
Patient Characteristics

Under the communication theme, subthemes were to tell or not to tell, whom to tell, and when to tell. Under the information seeking theme, subthemes were type of cancer, treatment options, and nature and management of adverse effects.

### Communication

All respondents identified communication as a key element of the physician-patient interaction. Ninety-five percent of patients stated that they wanted medical staff to tell them directly about their cancer diagnosis, whereas only 45% of family members wanted the patient to be told directly about the diagnosis. A young patient revealed how she learned about her cancer diagnosis by reading posters displayed on the walls of the hospital clinics. Statements of patients and their families were related to the three main questions (to tell or not to tell, whom to tell, and when to tell). Examples of patient and family statements related to the communication theme are shown in Table 2. Patients explained that in addition to the physician informing them of their diagnosis, the way they were told also had an impact on their ability to accept the news. Not every patient interviewed (5%); however, wanted to be told about their diagnosis directly. A 72-year-old patient with breast cancer, whose two daughters took care of her, wanted to hide the cancer diagnosis.

**Table 2:**
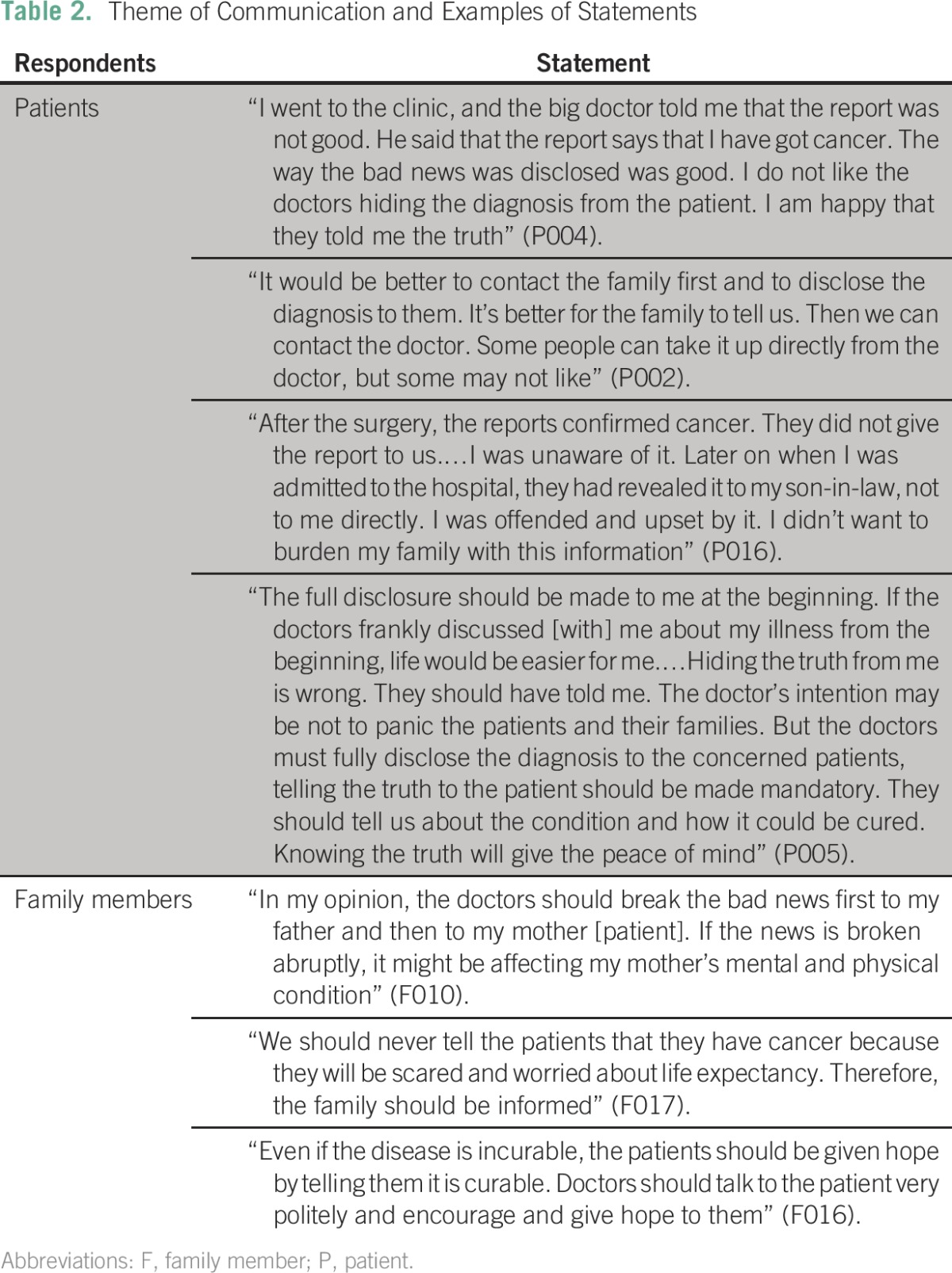
Theme of Communication and Examples of Statements

Fifty-five percent of the family members wanted medical staff to inform them of the cancer diagnosis, and they would then disclose the bad news to their family member if they deemed it necessary and/or appropriate. The most common reason family members wanted to be told of the diagnosis rather than the patient was fear that the patient would become upset. Family members wanted to protect their loved ones from such bad news and commonly spoke about the importance of medical staff giving patients hope, even in situations where the prognosis is likely to be poor. One family member spoke about her fears and difficulty in communicating with her husband’s treating physicians. She feared that her husband's treatment could be jeopardized as a result of their request for further information.

### Information Seeking

Information needs of the patients and families seemed similar regardless of differences in views on whom to tell. The majority of patients and families (80%) wanted specific information about the patient’s condition, including treatment options and medications, which was not given to them. The other 20% did not want to know details about the patient’s condition, treatment options, or prognosis because they believed that the physicians knew how to take care of the patient. Examples of statements of the respondents under the information seeking theme are shown in Table 3. Some patients reported that they had limited knowledge of chemotherapy and would have liked information about its effects, possible adverse effects, and steps they needed to take once they received chemotherapy. The majority of patients appreciated that information was disclosed to them in a staged approach rather than all at once. Some reported that they learned about cancer and available treatments from other patients on the oncology ward. Patients on the ward shared information about cancer and adverse effects of chemotherapy and seemed to support one another.

**Table 3:**
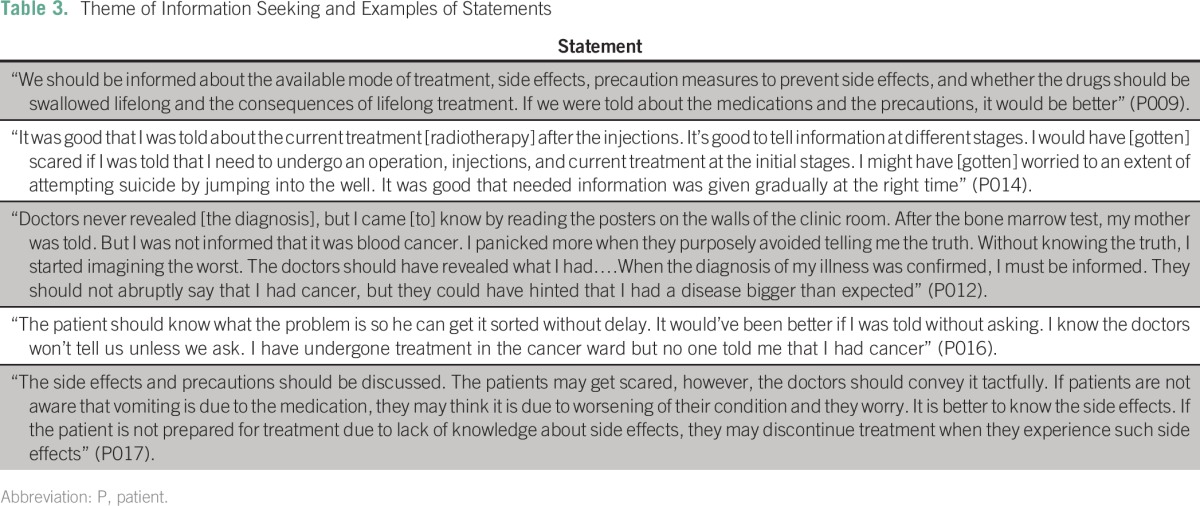
Theme of Information Seeking and Examples of Statements

## DISCUSSION

This study identified two major themes on the basis of patient and families’ perceptions about receiving bad news about a cancer diagnosis: communication and information seeking. Although the themes were the same for patients and families, the main difference was in the subtheme whom to tell. The majority (95%) of patients interviewed expressed a preference to be told directly about their cancer diagnosis. However, the majority of family members wanted the cancer diagnosis to be told to them mainly so that they could protect the patient from emotional harm. Patients and their families sought similar information relative to diagnosis, treatment options, and nature and management of adverse effects, although the required timing and quantity of information varied among respondents. This variation in requirement may reflect the educational levels of respondents, which can be addressed by offering written summary statements.

Results of this study are similar to a previous Sri Lankan study among Sinhalese that showed that the majority of patients wanted their diagnosis told directly to them, whereas the majority of the family members had opposing views without realizing the benefits of such direct disclosure of diagnostic and prognostic information.^18^ A similar situation existed in developed English-speaking countries in the 1960s, where a majority of physicians indicated a preference for not telling the truth to patients with cancer.^11^ However, this practice has changed rapidly during the past quarter of a century because of many factors, and breaking the bad news to the patient has become an important aspect of patient care.^12,15^ The main factors that influenced the changing trend of practice are a rise in patient autonomy in the physician-patient relationship, increasing complexity of diagnostic and therapeutic procedures that demand patient cooperation, development of trust between the patient and physician through honest communication, disclosure of the illness to give the patient an opportunity to adjust practically and emotionally to the illness, and that nondisclosure creates anxiety among patients.^14^

On the basis of the findings from the two Sri Lankan studies and experience from other non-Western countries and Western countries,^10,11^ our impression is that regardless of cultural background, the majority of patients want to know the truth. If the physician fails to inform, the patient will seek information from the Internet, other patients, and various other resources and may be bombarded with incorrect information. Such inaccurate and conflicting information can confuse the patient and create additional stress and anxiety.

Therefore, the main questions are how to break bad news in a busy clinical environment that is under-resourced in terms of workforce and space and how to change the culture in Sri Lanka and other developing nations. Solutions for cultural change may revolve around education (of families; medical, nursing, and health professionals; and society in general) about the importance of open communication, empowerment of patients, and training of health professionals in effective ways to break bad news. Through effective clinical leadership, society could be educated and empowered through public campaigns about breaking bad news to patients, such as through mass media (newspapers, magazines, and television).

Undergraduate and postgraduate curricula and assessment at medical schools and specialist colleges should include training in discussing prognosis/breaking bad news, respecting patient perspectives on the basis of cultural beliefs, and responding professionally. Because families are an integral part of the social fabric, they should be included in all management decisions and plans. Therefore, families should be educated during initial consultations on the need for and benefit of open disclosure of diagnosis and prognosis to patients. If the patient and family are too stressed during the first visit, subsequent consultations that gradually offer information may be needed.

Gaps in communication that arise from a limited medical workforce and space could be addressed by developing print materials in various languages; playing video clips in clinics, treatment waiting areas, and wards; and providing informal education sessions with nurses and other health professionals. Patients and families with higher emotional needs could be counseled in private areas, including physicians and nurses offices. Ultimately, multidisciplinary team members need to devise local solutions to respect privacy and confidentiality.

With regard to the Tamil diaspora, whether the same attitudes and practices remain is questionable. In many countries, several cancer centers have begun to produce printed materials in foreign languages, including Tamil. However, the majority of Tamils have lived in the Western world for many years and may have adapted to the culture and practices of the adopted countries. Similar studies among the large numbers of Sri Lankan Tamils living in Canada, the United States, Europe, and Australia would be informative.

How do oncologists in the Western world deal with breaking bad news to patients and families? Patients come from varying socioeconomic, ethnic, and educational backgrounds and have varying beliefs and needs; therefore, oncology treatment teams from the Western world have adopted a tailored approach by understanding patient preferences and perspectives, involving families, and using translators for effective communication.

Clinicians who are not familiar with qualitative methods might criticize this study. However, qualitative methods are appropriate to explore controversial topics and views of patients and families.^19^ In addition, we purposefully selected patients and families who had opposing views to explore their perspectives. A limitation of this study is that we did not ask questions about whether the cancer diagnosis, treatment, and information needs were related to religious beliefs.

In conclusion, this study shows that the majority of patients want to be told directly about their diagnosis and desire further information. An important first step would be the development of national guidelines for health professionals in breaking bad news and discussing prognosis by using the results of this and other studies. Future research could explore the connection between religious and cultural beliefs about life and death and patient and family information needs and acceptance of treatment modalities.
